# A network module-based method for identifying cancer prognostic signatures

**DOI:** 10.1186/gb-2012-13-12-r112

**Published:** 2012-12-10

**Authors:** Guanming Wu, Lincoln Stein

**Affiliations:** 1 Ontario Institute for Cancer Research, MaRS Centre, South Tower, 101 College Street, Suite 800, Toronto, ON M5G 0A3, Canada; 2 Department of Molecular Genetics, University of Toronto, 1 King's College Circle, #4386, Medical Sciences Building, Toronto ON M5S 1A8, Canada

## Abstract

Discovering robust prognostic gene signatures as biomarkers using genomics data can be challenging. We have developed a simple but efficient method for discovering prognostic biomarkers in cancer gene expression data sets using modules derived from a highly reliable gene functional interaction network. When applied to breast cancer, we discover a novel 31-gene signature associated with patient survival. The signature replicates across 5 independent gene expression studies, and outperforms 48 published gene signatures. When applied to ovarian cancer, the algorithm identifies a 75-gene signature associated with patient survival. A Cytoscape plugin implementation of the signature discovery method is available at http://wiki.reactome.org/index.php/Reactome_FI_Cytoscape_Plugin

## Background

A key goal in the application of genomics to disease is the identification of clinically relevant biomarkers that can distinguish otherwise indistinguishable patient subtypes. Laboratory tests based on these biomarkers can aid clinicians in identifying patients who are at higher risk of developing aggressive disease and thus would benefit from earlier, more aggressive therapy [1,2]. Biomarker-based tests can also guide clinicians in the choice of therapies most likely to benefit distinct patient groups [3-5].

Over the past decade, it has become clear that single biomarkers, such as the expression level of a particular protein, often do not perform as well as signatures created from ensembles of dozens or hundreds of genes, typically the expression levels of a panel of genes [6]. For example, Van de Vijver *et al. *[7] have built a classification system for breast cancer based on the gene expression profile of 70 genes, and found that their classifier outperforms standard systems based on clinical and histologic criteria. Pawitan *et al. *[8] have developed a 64-gene signature to predict the response to therapy of patients with breast cancer.

Several researchers have explained the observation that multi-gene signatures are more effective than single gene expression values by postulating that it is the network of gene interactions that underlies the phenotypes displayed by cells and that it is critical to understand normal and abnormal phenotypes through the lens of biological network perturbations [9]. In a recent review, Barabási, *et al. *[10] promoted network-based approaches for new cancer drug development and personalized medicine.

A variety of network-based analytic approaches of microarray gene expression data sets have recently been taken to search for gene signatures that are related to clinical outcomes in several cancers [11-16]. All of these are supervised algorithms in which the clinical parameter of interest, such as the disease-free survival of treated patients, informs the search for correlated network properties. The disadvantage of supervised algorithms is that they are prone to overtraining: a signature developed on one series of patients may fail to perform well on a different one.

Breast cancer is one of the leading causes of cancer death worldwide [17], and the most common cancer among women [18]. Expression of the cell surface protein biomarkers estrogen receptor (ER) and progesterone receptor (PR) have long been associated with a more favorable prognosis, while the presence of an amplification in the HER2/neu gene confers sensitivity to the targeted chemotherapeutic agent herceptin [19,20]. In recent years, breast cancer has become a favorite model for the development and testing of multi-gene prognostic signatures due to the heterogeneity in its clinical presentation and progression. Serous adenocarcinoma of the ovary is the leading cause of death from gynecological cancers in the United States, with over 22,000 new cases and 15,500 deaths per year [21]. In contrast to breast cancer, few independently replicated prognostic signatures exist for this disease [22].

In this paper, we describe a semi-supervised algorithm that first discovers modules of interacting genes involved in the disease process independently of clinical status, and then identifies clinically significant modules using the supervised principal component (superpc) method [23]. We apply this algorithm to breast cancer expression data to find a novel network module signature of 31 genes that is significantly related to breast cancer patient survival across five independent patient series. We then apply this method to a recent high-grade serous ovarian cancer expression data set to find a novel network module of 75 genes significantly correlated with patient survival. Both signatures outperform other well-known prognostic signatures as measured by statistical significance across independent data sets. The success of the technique on two very different tumor types suggests that it may be effective for other cancers as well.

## Results

Our method for discovering prognostic signatures builds on top of a human protein functional interaction (FI) network constructed by combining curated and uncurated data sources using a machine learning technique [24]. This FI network covers roughly half of annotated human proteins, and is highly reliable based on a variety of metrics, including confirmation of its predictions by domain experts [24]. The network as a whole is unweighted, and is not specific for any particular tissue or phenotype.

### Identifying disease-specific gene interaction modules

**Figure 1:**
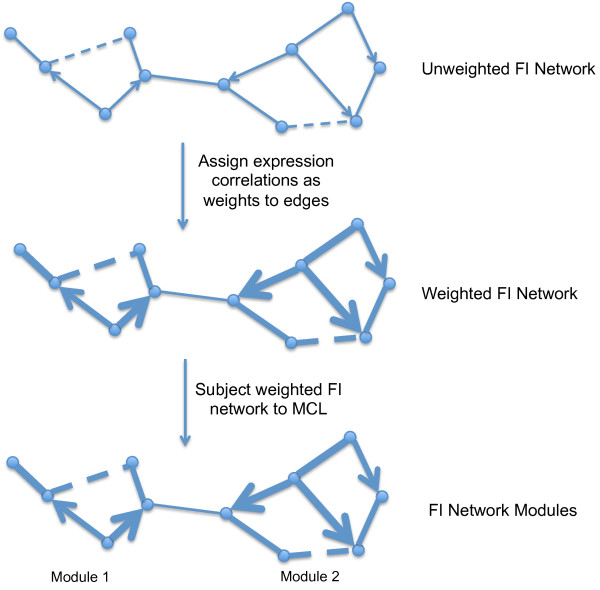
**Schematic workflow for network module searching based on gene expression data using a functional interaction network**.

The method applies to gene expression data sets in tissue samples from the disease of interest (Figure 1), typically, but not necessarily, obtained via expression microarray. We first calculate the Pearson correlation coefficients (PCCs) among all functional interaction pairs in the gene expression data set. We then assign the PCCs to the edges of the FI network, thereby converting an unweighted generic graph into a weighted disease-specific graph. Next, we use a highly efficient network clustering algorithm, MCL (Markov clustering) [25], to cluster the weighted network into a series of gene interaction modules. Each module consists of a set of genes that are both topologically close in the tissue-agnostic protein FI network, and highly correlated by expression level in the disease-specific expression data set.

The MCL step generates many network modules, the majority of which are very small, and contain two or three genes only. We filter the modules passed to the next step of analysis by removing those that are smaller than an arbitrary threshold *n*, and which have an average PCC below a second arbitrary threshold *p*. We typically select *n *= 8 and *p *= 0.25.

From the filtered list of modules, we create a module-based gene expression matrix with the original expression data set, which provides a mean expression level for each module across each tissue in the series. We then apply this expression matrix for the superpc analysis [23] to search for linear combinations of network modules that are significantly correlated with patient survival or other clinically relevant criteria.

### Discovery and validation of candidate prognostic modules in breast cancer

Our approach is divided into two phases: prognostic module discovery and validation. For module discovery, we used a breast cancer tissue microarray expression data set published in 2002 [7], henceforward known as the 'Nejm' set. For validation, we used a total of four independent breast cancer microarray expression data sets (Table 1). The Nejm set contains gene expression data and clinical information for samples derived from 295 patients with primary invasive breast carcinomas, and the four validation data sets contain samples derived from between 158 and 372 patients each, for a total of 1,233 independent patients. Notably, different microarray platforms were used for each of the discovery and validation sets, except for GSE4922 [26] and GSE1456 [8], which are composed of non-redundant tissue samples analyzed on the same platform. **{**In each patient series, survival was defined as the number of years or months from initial pathological diagnosis of the tumor until patient demise or loss to follow-up. However, the time durations for GSE4922 are for disease free survival times since only disease free survival times are provided in this data set.

**Table 1:** Five breast cancer data sets used in this study

Data set	Sample size	Cancer type	Microarray platform	Reference
Nejm	295	Primary invasive breast carcinoma	Agilent Hu25K	[7]
GSE1456	159	Primary breast cancer	Affymetrix Hu133A, Hu133B	[8]
GSE18229	372	Invasive breast carcinoma	Agilent 1A, Agilent custom array	[64]
GSE3143	158	Primary breast tumor	Affymetrix Hu95Av2	[65]
GSE4922	249	Primary breast cancer	Affymetrix Hu133A, Hu133B	[26]

The Nejm data set was used as the training data set for searching for network modules that are clinically significant, and the other four data sets were used as test data sets.

In the discovery set, 30 modules ranging in size from 8 to 70 genes passed the filters. We numbered these from 1 to 30 in order of decreasing module size. Based on these modules, we generated a module-based gene expression matrix by using mean expression level for each module across 295 breast cancer samples, and subjected this matrix to superpc analysis. Superpc ten-fold cross-validation [23] results show an optimal threshold value at 1.20, which yields 8 modules comprising 165 genes. We then validated the trained superpc model based on the selected network modules against four independent breast cancer data series. The results (Table 2) show that the same first principal component is significantly related to patient overall survival across five breast cancer data sets with *P*-values ranging from 4.1 × 10^-3 ^to 6.7 × 10^-11^.

**Table 2:** Superpc continuous prediction results from breast cancer data analysis

	Principal component		
	
	1st	2nd	3rd
NEJM			
HR	5.21E+00	2.28E+00	1.98E+00
95% HR CI	3.18 to 8.56	1.19 to 4.34	1.02 to 3.83
*P*-value	**6.68E-11**	**1.25E-02**	**4.26E-02**
GSE4922			
HR	7.35E+00	2.05E-01	2.66E-02
95% HR CI	2.11 to 25.6	0.0091 to 4.62	6.5e-4 to 1.08
*P*-value	**1.73E-03**	3.19E-01	5.51E-02
GSE3143			
HR	7.27E+02	6.10E-03	1.70E+00
95% HR CI	8.1 to 6.5e+4	1.8e-4 to 0.21	1.1 to 2.6
*P*-value	**4.12E-03**	**4.61E-03**	1.19E-02
GSE18229			
HR	5.34E+00	7.20E+00	9.31E-01
95% HR CI	2.63 to 10.9	1.73 to 30.0	0.30 to 2.90
*P*-value	**3.63E-06**	**6.66E-03**	9.01E-01
GSE1456			
HR	1.50E+02	1.45E+02	6.03E+00
95% HR CI	17 to 1287	1.66 to 1.26e+4	0.034 to 1057
*P*-value	**5.00E-06**	**2.91E-02**	4.96E-01

The results were generated by using the Nejm data set as the training data set and four independent data sets as validation data with a threshold value of 1.20 and 8 selected MCL modules. *P*-values less than 0.05 are in bold. CI, confidence interval; HR, hazard ratio. **{**

In order to assess the robustness of our signature-finding algorithm, we used each of four validation data sets as the training data set, and the remaining four as the validation data sets (Tables S1 to S4 in Additional file 1). Except for results related to GSE3143, all trained superpc models yielded highly similar prognostic modules as measured by gene overlap: using a hypergeometric test for the statistical significance of the overlap yielded *P*-values ranging from 1 × 10^-11 ^to 1.7 × 10^-78 ^(Table S5 in Additional file 1).

When applied to GSE3143, the training procedure selected four MCL modules that contained a distinct set of genes from those selected by the other data sets, and yielded much wider 95% confidence intervals on the hazard ratios derived from these modules (Table S2 in Additional file 1). This may result from poor quality of this data set, a different patient or tumor population, or unknown factors. However, the trained superpc model using GSE3143 as the training data set remained significant across all five data sets (Table S2 in Additional file 1).

MCL can be used for finding network clusters based on the original unweighted, tissue-agnostic FI network. By assigning gene co-expression values as weights for the FI network, we hope to find network modules containing genes having similar expression patterns in a disease, and expect these modules are better features to model disease heterogeneity. We compared the weighted approach to an unweighted approach and found that the weighted approach has better performance (Table S6 in Additional file 1) based on both R^2 ^values, which are used to measure the performance of survival models [23], and *P*-values. The unweighted approach also failed to yield significant results for the ovarian cancer data sets (see below).

### Module 2 is significant across five breast cancer data sets

Our earlier results show that training with the Nejm data set yields a superpc model with the best *P*-values across all validation sets. We therefore investigated the structure of this signature in more detail by assessing the contribution of each individual network module to the signature's superpc model.

Using the univariate Cox proportional hazards (Cox PH) model [27,28] to measure the correlation of mean expression level of each of eight modules selected by the trained superpc model to patient survival time (Table 3), we found that each module is correlated with patient overall survival at *P*-values ≤ 0.002. However, the most striking of these was module 2, a set of 31 genes with a hazard ratio of 1.3 and having the lowest *P*-value of 1.75 × 10^-10^. This *P*-value remains significant after strict Bonferroni correction (27 tested MCL modules, adjusted *P*-value 4.7 × 10^-9^). By repeating the Cox PH analysis on each validation data set, we found that module 2 alone is consistently correlated with survival in all four of the sets (Table 3). Furthermore, it has the lowest *P*-values in four validation data sets, ranging from 1.64 × 10^-4 ^in GSE18229 to 6.70 × 10^-6 ^in GSE3143. For this reason, we examined module 2 in more detail.

**Table 3:** Uni-variate Cox proportional hazards analysis results for right Markov clustering modules

		Nejm		GSE4922		GSE3143		GSE18229		GSE1456	
		
Module	Size	Hazard ratio	*P*-value	Hazard ratio	*P*-value	Hazard ratio	*P*-value	Hazard ratio	*P*-value	Hazard ratio	*P*-value
2	31	**1.30E+00**	**1.75E-10**	**1.84E+00**	**5.84E-05**	**1.55E+01**	**6.70E-06**	**1.12E+00**	**1.64E-04**	**2.46E+00**	**8.76E-05**
18	9	9.44E-01	3.99E-09	1.35E+00	1.86E-04	2.61E+00	1.42E-01	7.23E-01	2.59E-03	2.19E+00	1.10E-04
4	17	1.14E+00	1.35E-08	2.46E+00	2.95E-04	2.18E-01	9.23E-01	1.08E+00	3.81E-03	3.38E+00	8.42E-04
13	11	1.02E+00	7.49E-07	1.84E+00	2.02E-04	6.20E-01	3.28E-01	8.66E-01	3.31E-04	2.51E+00	2.37E-04
21	8	1.75E+00	5.21E-05	2.08E+00	6.61E-02	-2.14E+00	2.48E-01	1.31E+00	3.44E-02	3.06E+00	2.93E-02
12	11	-6.59E-01	3.59E-04	-7.18E-01	3.40E-01	3.07E+00	8.43E-02	-8.81E-01	1.26E-02	-3.32E+00	1.55E-02
29	8	7.90E-01	9.21E-04	1.80E+00	5.03E-02	-1.08E-01	4.89E-01	1.25E+00	2.41E-03	3.84E+00	2.58E-03
1	70	-6.68E-01	2.05E-03	2.96E+00	2.95E-02	-1.41E-01	2.62E-01	-1.75E-01	6.38E-01	-2.04E+00	2.76E-01

The eight MCL modules were selected by the trained superpc model when the Nejm data set was used as the training data set. Modules are sorted based on *P*-values. The sole module that is significant across five breast cancer data sets is in bold.

**Figure 2:**
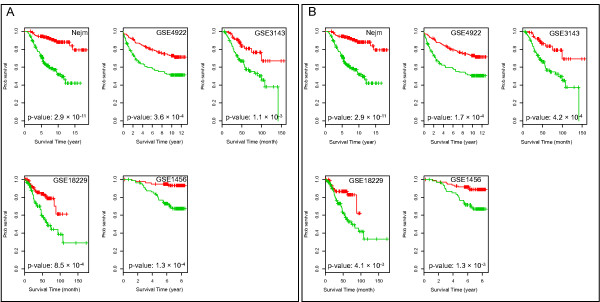
**Kaplan-Meier survival plots for the five breast cancer data sets**. Samples in each data set were split into two groups using the median values as the cutoff values. Green, samples having higher module 2 expression; red, samples having lower module 2 expression. Note that the time durations for GSE4922 are for disease free survival times. **(a) **Results before rescaling module 2 expression values across five breast cancer data sets. **(b) **Results after rescaling.

We partitioned samples in each data set into two groups corresponding to high and low module 2 expression based on the median expression level of genes in the module and generated Kaplan-Meier survival curves (Figure 2). In each case, high module 2 expression is associated with significantly shorter survival.

**Figure 3:**
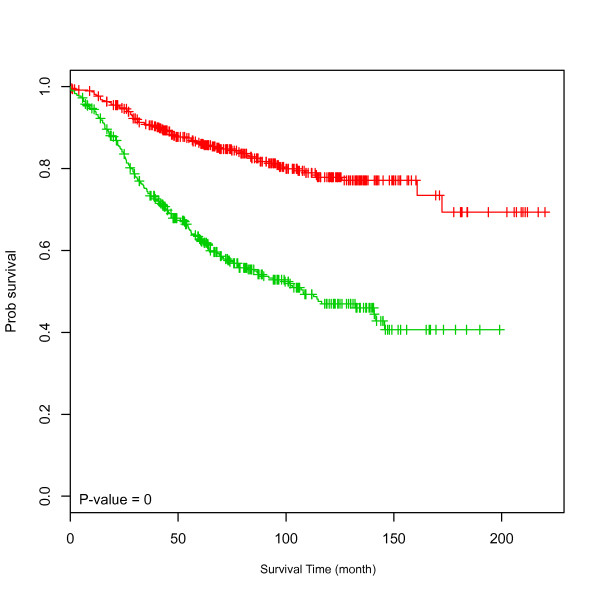
**Kaplan-Meier survival plot for the merged breast cancer data set**. All samples were divided into two groups based on the median value of module 2 expression. The green curve is for samples having higher module 2 expression, while the red curve for samples having lower expression.

Because the studies used diverse array platforms, the absolute expression score for module 2 that we chose as the pivot point differed among the studies. The need to calculate the pivot point from a population of patient samples is an obstacle to using module 2 as a potential prognostic test. However, we found that a simple procedure for rescaling each of the array platforms to match the profile of the Nejm set (Figure S2 in Additional file 1) allowed us to directly compare module expression levels across arrays and to merge all samples from the discovery data series and all four validation series into a single series of 1,233 patient samples. As shown in Figure 3, the merged data set shows strikingly different survival curves among the high and low module 2 expression groups, and acts as a proof of principle for using module 2 expression as a cross-platform prognostic signature.

It has been reported that microarray-based gene signatures are effective in ER+, but not in ER- breast cancers [1]. We looked at the special case of triple negative (TN) samples, which are negative for ER and PR as well as HER2/neu amplifications, and have a poor prognosis overall. For this analysis, we only used the GSE18229 data set since it is unique in providing information about ER, PR and HER2 status. Figure S3 in Additional file 1 shows Kaplan-Meier survival curves for the four sample groups: ER+/Module2+, ER+/Module2-, TN/Module2+, and TN/Module2-. Among ER+ patients, there is a striking difference in survival between Module2+ and Module2- patients. Among TN patients, there is no difference in survival for the first 30 months, but after this time there is a paradoxical increase in survival in patients with Module2+ tumors. However, this difference is near the borderline of significance, at *P*-value = 0.088, and the number of patients is small (*n *= 59). Therefore these results should be interpreted with caution.

As a check of the statistical significance of module 2, we performed a permutation test on the Nejm data set by repeatedly swapping gene expression values. In 1,000 permutations, we never found a network module with a *P*-value below 1.64 × 10^-4^, the highest *P*-value, across all five breast cancer data sets. We also performed a permutation test using gene sets randomly selected from the FI network and containing the same number of genes as module 2, and failed to find a random gene set with *P*-value below 1.64 × 10^-4 ^across five data sets after 1,000 trials. These results indicate that module 2 was highly unlikely to have been found by chance.

### Biological role of module 2

**Figure 4:**
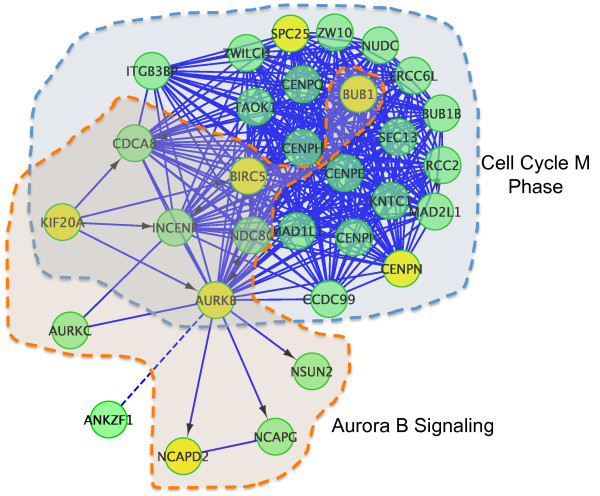
**Functional interaction sub-network constructed by using module 2 genes**. Gene products involved in the Aurora B signaling pathway have been encapsulated in the light brown shaded area, while gene products involved as components of the kinetochore in mitosis in the light grey shaded area. Edges between genes indicate functional interactions (FIs). FIs extracted from pathways are shown as solid lines (for example, AURKB→NCAPD2), while predicted ones are shown as dashed lines (for example, AURKB---ANKZF1). Extracted FIs involved in activation, expression regulation, or catalysis are labeled with arrows. Seven genes highlighted in yellow form a smaller network module that is also significantly related to breast cancer patient survival across five patient data sets.

To understand why the 31 genes comprising module 2 might be related to patient survival, we performed a functional enrichment analysis on this module and its subnetworks using pathways that were used to build the FI network (Figure 4). There are two interdigitating subnetworks in module 2, one enriched in genes annotated as 'Cell Cycle M Phase' in Reactome [29], and the other as 'Aurora B Signaling Pathway' in the NCI-PID database [30]. An enrichment analysis on Gene Ontology cellular component annotations indicates a strong enrichment in gene products involved in condensed chromosome kinetochore or centromeric regions (Table S8 in Additional file 1).

We also tested if it is possible to find a smaller subset of genes from this module. After sorting module 2 genes based on *P*-values generated from the univariate Cox PH model (Table S9 in Additional file 1), we chose the top genes using different *P*-value cutoffs. **{**Using this approach, we found that smaller subsets of genes are also significant across five breast cancer data sets. For example, with a *P*-value cutoff 1.0 × 10^-7^, we identify seven genes (highlighted in yellow in Figure 4): *ARUKB*, *BIRC5*, *BUB1*, *CENPN*, *KIF20A*, *NCAPD2*, and *SPC25*. The *P*-values generated from Cox analysis range from 2.0 × 10^-4 ^to 1.2 × 10^-11 ^for the five studies. Pathway enrichment analysis on this smaller set of genes was similar to the results described for the entire module 2.

### Comparison between module 2 and other breast cancer prognostic gene signatures

**Figure 5:**
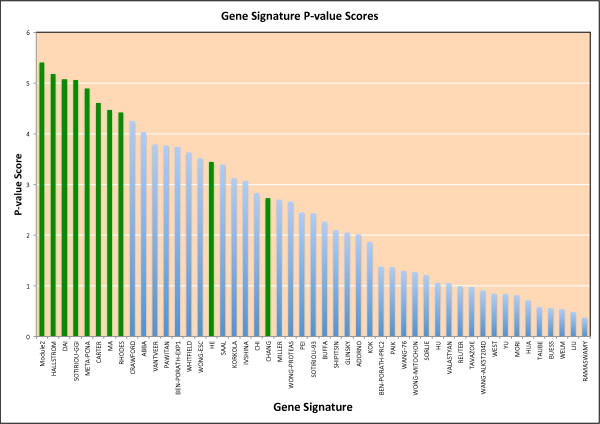
**Plot of *P*-value scores for module 2 and 48 published breast cancer gene signatures**. Published gene signatures were collected and reported by Venet *et al. *[31]. The *P*-value score is defined as the negative logarithm of the geometric average of *P*-values from univariate Cox PH survival analyses from five breast cancer data sets. Signatures having *P*-values less than 0.05 across all five breast cancer data sets are plotted in green.

Many breast cancer prognostic gene signatures have been published since the first 70-gene signature was developed about 10 years ago [1]. We compared the correlation significance of average gene expression and breast cancer patient overall survival between our module 2 and 48 gene signatures collected and reported by Venet *et al. *[31] across five breast cancer data sets. In order to compare the gene signature performance across all five breast cancer data sets, we used the negative logarithm of geometric mean of *P*-values from five breast cancer data sets based on the univariate Cox PH model as the score (*P*-value score). The higher this score, the better a gene signature performs. Figure 5 is the plot of *P*-value scores for module 2 and 48 published gene signatures, and shows that module 2 has the best performance across five breast cancer data sets. For any individual data set, however, the module 2 signature never has the top ranked *P*-value (Figure S5 in Additional file 1). We take these results as evidence that the semi-supervised nature of the signature discovery algorithm leads to a gene expression signature that is more robust than those discovered by supervised methods.

Venet *et al. *[31] found that many published breast cancer gene signatures have strong expression correlation to a cell proliferation-related gene set called meta-PCNA, containing 131 genes that are significantly related to PCNA gene expression. We checked the gene expression correlation between module 2 and meta-PCNA, and found that similar to other many published gene signatures, module 2 expression is strongly correlated to meta-PCNA across five breast cancer data sets (Table S13 in Additional file 1). Because of this strong correlation, multi-covariate Cox PH analysis shows that module 2 is not independently related to patient overall survival after adjustment for meta-PCNA gene expression in all but the GSE3143 data sets (Table S14 in Additional file 1). Gene overlap analysis shows that 7 out of 31 genes in module 2 are shared with meta-PCNA (*P*-value = 3.5 × 10^-10^). All the shared genes (*BIRC5*, *AURKB*, *KIF20A*, *NCPAD2*, *BUB1*, *CDCA8*, and *MAD2L1*) are among the top ten of module 2 genes sorted by *P*-values based on the NEJM data set (Table S9 in Additional file 1). These results again point to a biological role in cell proliferation for module 2.

### Application of the method to high-grade serous adenocarcinoma of the ovary

To test our method in another cancer type, we applied it to high-grade serous adenocarcinoma of the ovary. We used The Cancer Genome Atlas (TCGA **{**) ovarian gene expression data set [32] as the training data set, and three independent data sets (GSE9891 [33], GSE13876 [34], GSE26712 [35]) for validation.

The primary MCL network clustering algorithm identified 27 modules of size 8 or greater in the TCGA training set, 7 of which were selected by superpc using a threshold value of 0.8. Using superpc's continuous prediction model, we found that the second principal component is significantly related to ovarian cancer patient overall survival across four independent ovarian cancer data sets (Table S15 in Additional file 1). Superpc also offers a 'discrete prediction' model in which patient samples are divided between two groups based on the training data set. A univariate Cox PH analysis (Table 4) and Kaplan-Meier plot (Figure S6 in Additional file 1) demonstrate that the second principal component distinguishes a subgroup of patients who have significantly longer survival (hazard ratio (HR **{**) ranging from 1.33 to 2.43 among the four data sets, *P*-values ranging from 3.61 × 10^-2 ^to 8.85 × 10^-5^). Both results show that the second principal component can be used as a feature to predict samples into two groups: one contains samples having longer survivals, while another shorter survivals.

**Table 4:** Superpc discrete prediction results

**Data set**	Prediction results
TCGA	
HR	1.33E+00
95% HR CI	1.04 - 1.69
*P*-value	**2.21E-02**
GSE9891	
HR	2.43E+00
95% HR CI	1.06 - 5.55
*P*-value	**3.61E-02**
GSE13876	
HR	2.13E+00
95% HR CI	1.46 - 3.10
*P*-value	**8.85E-05**
GSE26712	
HR	1.56E+00
95% HR CI	1.08 - 2.25
*P*-value	**1.75E-02**

The TCGA data set was used as the training data set, and the second principal component was used as the feature for prediction. The first column shows the data sets. CI, confidence interval; HR, hazard ratio.

In contrast to our results in breast cancer, we failed to find an individual module that was significantly related to patient overall survival across the training set and all three validation sets. So we used the six network modules selected by superpc and built a functional interaction subnetwork of 75 genes (Figure S7 in Additional file 1). Pathway annotations show that the majority of these modules are related to interleukin 2 and T-cell receptor signaling transduction pathways, cell adhesion, cell cycle, and homologous recombination.

Recently, Mankoo *et al. *[22] used the same TCGA data set to identify prognostic signatures for progression-free survival (PFS; 181 genes) and overall survival (219 genes) in ovarian cancer using information from copy-number alteration, mRNA expression, microRNA expression, and RNA methylation data. We compared their mRNA overall survival signature to the 75-gene signature derived from MCL+superpc. In comparison to the Mankoo signature, the MCL+superpc signature is almost one-third of its size, and performs better across the same three ovarian cancer data sets used. **{**The Mankoo *et al*.'s mRNA signature yields *P*-values ranging from 0.18 to 0.014 (Kaplan-Meier plot using t-score **{**; Figure 1 SC in File S1 in [22]), or from 0.71 to 0.048 (Kaplan-Meier plot using c-score **{**). In contrast, the MCL+superpc signature shows *P*-values from 0.036 to 0.017, which are significant across all data sets.

### Cytoscape plug-in

We have packaged the workflow of module discovery, and survival analysis, except the superpc analysis, into a software tool that runs within a popular biological network analysis platform, Cytoscape [36]. This tool is called the Reactome FI plug-in and can be downloaded from [37]. We also provide a detailed user guide and example data sets. The plugin operates by making RESTful requests against Java and R server-side services that reside at the Reactome server. The code underlying the plugin and server-side services is available under an open source license that allows for unrestricted use, modification and redistribution.

## Discussion

In this paper we describe a simple and rapid procedure to combine disease-specific gene expression data with a static protein functional interaction network in order to identify candidate prognostic network modules. We apply this method to breast cancer, and find a network module enriched in Aurora B signaling and kinetochore components, whose expression is strongly anticorrelated with breast cancer patient survival. The network module was validated in four independent data sets, across a total of more than 1,200 patients.

It is widely believed that modular structures exist in protein interaction networks and other biological networks [10]. In his recent review, Barabási [10] proposed that three types of network modules can be extracted from protein interaction networks: topological modules, functional modules and disease modules. Topological modules are sets of interacting proteins that tend to link to each other instead of to others; functional modules are graph components that confer specific cellular functions; and disease modules are graph components underlying one or more diseases. Our approach links these three kinds of modules together by using MCL network clustering to detect topological modules in a functional interaction network weighted by disease-specific expression data. By weighting the edges in the FI network using gene co-expressions, we find network modules comprising genes having similar expression profiles in a disease. At least in the breast cancer model, weighted MCL modules yield more robust signatures than unweighted modules, implying that the weighted modules catch network patterns related to heterogeneity in a disease.

The candidate breast cancer prognostic module that we have found contains 31 genes and combines components of Aurora B kinase signaling, the cell cycle M phase, and kinetochore maintenance. Uncontrolled cell proliferation is the major phenotype displayed by cancer cells [38] and multiple studies have connected increased metrics of mitotic activity to poorer patient survival in breast cancer [39-41]. This is consistent with our observation that increased expression among the genes comprising the module is associated with poorer overall patient survival. The strong correlation between meta-PCNA [31] and module 2 also implies that proteins in module 2 are related to cell proliferation. The majority of currently published gene signatures are related to cell proliferation too [1,31]; nevertheless our module 2-based signature is more robust, and performs better than those signatures across five breast cancer data sets, including more than 1,200 patients in total.

The aurora kinases are a family of serine-threonine kinases that are key regulators of mitosis and many signaling pathways, and evidence is accumulating that these proteins play an important role in the malignant cell cycle [42]. There are three members of this family in human: Aurora kinase A (AURKA), B (AURKB), and C (AURKC). Currently, most research efforts have been focused on AURKA protein, and evidence indicates that AURKA expression, but not AURKB expression, is predictive of patient survival in breast cancer [43]. **{**The module we have found contains both AURKB and AURKC, but not AURKA. Survival analysis using the univariate Cox PH model with single gene expression shows that AURKB, but not AURKC, expression is significantly related to patient survival in five breast cancer data sets (Table S9 in Additional file 1). Furthermore, one of AURKB's functions in human cells is to ensure proper kinetochore-microtubule attachments [42], raising the possibility that the signature we have identified may act by virtue of its effect on kinetochore maintenance in addition to, or instead of, its effect on mitotic rate.

We observe that breast cancer module 2 expression is strongly correlated with ER status (Table S12 in Additional file 1). The Reactome functional interaction network in fact does predict an interaction between ESR1 and ITBG3BP, a member of module 2. Talukder *et al. *[44] showed that *ITBG3BP *is an estrogen inducible gene; ITBG3BP protein also associates with endogenous ER and acts as a transcriptional co-regulator of ER target genes [44]. ITBG3BP has also been found to be involved in assembly of a proximal CENP-A nucleosome associated complex (NAC), which is essential to the function of the kinetochore to maintain correct chromosome alignment and segregation during mitosis [45]. This suggests a direct connection between ER status and module 2 expression, but contradictory to our finding of anti-correlation between module 2 expression and ER level. Besides this direction connection between ESR1 and module 2, there are many indirect connections via FIs in the FI network. We will analyze these indirect connections to see why there is anti-correlation between module 2 expression and ER level.

Network-based approaches are different from gene based approaches [7,8,46]. In network based approaches, protein interaction networks are used in gene signature search. Genes in found signatures usually interact with each other, are involved in the same pathways, and have similar biological functions. Chuang *et al. *[14] showed that network-based approaches perform better in cancer metastasis prediction than gene-based approaches. Similar to what Chuang *et al. *[14] showed before, our network module-based approach shows more consistent performance across different data sets than the gene-based approach in the superpc analysis.

Several methods have been published for using protein interaction networks to search for clinically relevant network components, subnetworks or modules [14-16,47]. The most popular one is the greedy search algorithm [47]. Our weighted MCL network module search method runs much faster than the greedy based network module search approach: 20 second versus 6 hours (see the supplementary results in Additional file 1 for details). During greedy search, the majority of running time has to be spent calculating Cox PH scores for each potential network module in order to find final modules. However, there is no need to run CoxPH survival analysis in MCL network clustering.

Currently, all published network module search methods work in a supervised manner in which the phenotype information is used during the component search process as all gene-based approaches [7,8,46] do. We use an unsupervised approach for module construction in which the only inputs are gene coexpression values and the static functional interaction network. We believe this use of an unsupervised approach contributed to the success of validating the candidate prognostic module against multiple independent data sets. Indeed, the supervised greedy search algorithm shows a serious over-training problem, with much bigger performance differences based on *P*-values between the training data set and the testing data sets (Tables S1 and S7 in Additional file 1). We observed similar over-training issues when we applied linear regression methods to MCL network modules (data not shown).

Horvath and colleagues [48,49] have developed a framework for network-based analysis using a weighted correlation network based on expression data sets. Our approach differs from theirs in that they construct the network based on gene expression correlation directly, while we use a pre-built static functional interaction network. In the Horvath methodology, the edges between two genes in the weighted co-expression network may not necessarily mean direct functional relationships, while those built from functional interactions were built from a combination of human curated pathways and predicted interactions supported by multiple independent data sources. We believe this increases the likelihood that the candidate module-based signatures we find reflect true functional units in the cell.

The application of our MCL+superpc approach to high grade serous adenocarcinoma of the ovary identified 6 modules comprising 75 genes that appear to be associated with patient overall survival in ovarian cancer. These modules are related to immune system function, cell adhesion, and cell cycle activity. Several groups have published ovarian cancer signatures based on microarray expression profiles. Most, if not all, of these signatures were developed using individual genes without considering network context during signature search. Many of the studies had small sample sizes [35,50,51], or were not validated using independent data sets [51]. Very recently, however, Mankoo *et al. *[22] used the same data sets that we use here to study signatures for both progression-free survival and overall survival in ovarian cancer. These authors integrated features from copy-number alteration, mRNA expression, microRNA expression, and RNA methylation data. Even so, the signature discovered by our MCL module-based method combined with superpc outperformed theirs across the three shared ovarian cancer data sets, suggesting that simple gene expression values, when combined with *a priori *network information, can yield prognostic signatures comparable to those derived by combining diverse molecular features.

## Conclusions

We report a new network-based approach to identify functional interaction modules in gene expression data sets, used it to find a novel 31-gene signature that is strongly correlative with poor patient survival in ER-positive breast ductal adenocarcinoma patients, and validated the signature in five independent patient series. This network module is related to Aurora kinase B signaling and kinetochore assembly during mitosis. Using this approach, we have also identified a 75-gene signature that is correlated with survival in high-grade serous adenocarcinoma of the ovary. We have packaged the algorithms in a software package that is available for unrestricted use and redistribution.

## Materials and methods

### Functional interaction network construction

The functional interaction network used in this study was described in Wu *et al. *[24]. Briefly, we compiled protein pairwise relationships extracted from protein-protein interactions from human, yeast, worm, and fly, gene co-expression data sets, Gene Ontology annotations, domain-domain interactions, and text-mined protein interactions. We then trained a naïve Bayes classifier based on these pairwise relationships by using a training data set extracted from curated pathways from Reactome [29]. The trained naïve Bayes classifier was used to predict functional interactions for protein pairs, and the predicted FIs merged with FIs extracted from curated pathways in Reactome, KEGG [52], NCI-PID [30], Panther [53] and CellMap [54]. The final FI network contains 10,956 proteins, and 209,988 interactions.

### MCL network clustering

We chose MCL [25] as the network-clustering algorithm in order to take advantage of edge weights. We weighted each interaction edge according to the absolute value of the PCC of the expression levels of the two genes connected by the edge. To control the size of network modules generated from the MCL clustering, we used 5.0 as the inflation coefficient. For breast cancer data analysis, we chose MCL modules of size 8 or greater, and average PCC no smaller than 0.25. In the breast cancer data set, these two filters yielded 30 MCL modules comprising 401 genes. For permutation testing, we randomly swapped expression values for all genes, or randomly selected genes from the FI network.

### Greedy network module search

Following the method described in Chuang *et al. *[14], we implemented a greedy network component search method in Java to search for network modules significantly related to patient overall survival. We used a Java package called JavaStatSoft [55] to do Cox PH survival analysis, and used the negative logarithm of *P*-values from the Cox PH model as component scores during network searching. The search depth was 1 and max depth was 2.

### Superpc analysis

For the superpc analysis, we used the R package superpc downloaded from CRAN [56], and followed the instructions on the authors' web site [57]. To use the second principal component as the feature for discrete prediction in the ovarian cancer data analysis, we modified the original R source code. For gene-based superpc analysis for the breast cancer data sets, we grouped a set of 1,849 genes that are shared across all five breast cancer data sets. For the ovarian cancer data analysis, we did a z-score transformation on MCL module-based gene expression matrices.

### Breast cancer data sets

The five breast cancer data sets were downloaded from published sources. The Nejm [7] data set was downloaded from the authors' web site [58]. It contains a gene expression data set and clinical information for 295 primary breast cancer patients. The other four breast cancer data sets, which were used as validation data, were downloaded from GEO [59]. See Table 1 for detailed information. We downloaded SOFT formatted family files, extracted clinical information, and mapped probes to genes based on annotations in the downloaded files using an in-house Java parser. Probes that could be mapped to multiple genes were removed. In cases in which multiple probes were mapped to the same gene, we used averaged values for multiple probes as the gene expression value. All gene expression data sets have been z-score transformed as described in [60] before further analyses. To classify breast cancer samples in the data sets, we used the downloaded R code for the PAM50 classifier from [61].

### Ovarian cancer data sets

Four ovarian cancer data sets have been used. The TCGA ovarian cancer data set [32] was provided by the TCGA analysis group. The other three data sets, GSE9891 [33], GSE13876 [34], and GSE26712 [35], were downloaded from GEO, and were pre-processed as for the breast cancer data sets.

### Survival analysis

We used R [62] for both the Cox PH model [27] and Kaplan-Meier survival analysis [28]. *P*-values reported in Results were based on the Wald test for the Cox PH model, and log-rank test for Kaplan-Meier analysis. To assign an expression score to a module generated from the MCL clustering, we took the mean gene expression value for all genes contained in the module.

### Reactome FI Cytoscape plug-in development

We have implemented the signature discovery procedures described here as a Cytoscape plug-in [36], which uses a lightweight client and server two-tier architecture. The major analysis functions were implemented in the server side and exposed in RESTful APIs. The client-side Cytoscape plug-in is used to display the results returned from the server-side. This plug-in has been configured so that it can be launched via Java Web Start [63], or can be installed by downloading a jar file and placed into the plug-in folder in the Cytoscape application. The source code can be downloaded by following a link from the user guide page [37].

## Abbreviations

Cox PH: Cox proportional hazards; ER: estrogen receptor; FI: functional interaction; MCL: Markov clustering; PCC: Pearson correlation coefficient; PR: progesterone receptor; superpc: supervised principal component; TCGA: The Cancer Genome Atlas **{**; TN: triple negative.

## Authors' contributions

GW designed the study, collected data sets, developed the algorithm, performed data analyses, and wrote the manuscript. LS supervised the project, participated in data analysis, and wrote the manuscript. Both authors have read and approved the final manuscript.

## Supplementary Material

Additional file 1 **Supplementary results**. Click here for file
