# Supplementary material for: A network module-based method for identifying cancer prognostic signatures
Source: Genome Biol. 2012 Dec 10;13(12):R112. doi: 10.1186/gb-2012-13-12-r112 (PMC3580410; doi:10.1186/gb-2012-13-12-r112)
Supplement: Additional file 1 — Supplementary results. [file gb-2012-13-12-r112-S1.PDF]

Supplementary Results

Results from using different breast cancer data set as the training data set in the MCL+superpc approach

In order to check the robustness of our MCL+superpc approach, we used each of four validation data sets as the training data set, and the remaining four data sets as validation data sets. The following tables show these results.

Table S1. *Superpc continuous prediction results from breast cancer data analysis. The results were generated by using the GSE4922 data set as the training data set and four independent data sets as validation data with a threshold value of 1.10 and 9 selected MCL modules. The training data set is highlighted in red. P-values less than 0.05 are highlighted in yellow.*

**Principal Component (PC)**		**1st**	**2nd**	**3rd**	**Overall**
	**HR**	5.27E+00	1.78E-01	1.22E+00	
**NEJM**	**95% HR CI**	3.02 - 9.2	0.062 - 0.51	0.55 - 2.73	R^2: 0.129
	**p-value**	5.07E-09	1.36E-03	6.23E-01	4.37E-09
	**HR**	3.31E+01	9.06E+00	1.44E+00	
**GSE4922**	**95% HR CI**	6.97 - 157	0.23 - 365	0.036 - 57	R^2: 0.076
	**p-value**	1.07E-05	2.42E-01	8.46E-01	2.03E-04
	**HR**	5.04E-01	8.64E-01	1.12E+02	
**GSE3143**	**95% HR CI**	3.2e-4 - 785	1.1e-3 - 665	4.6 - 2705	R^2: 0.093
	**p-value**	8.55E-01	9.66E-01	3.73E-03	5.12E-03
	**HR**	3.87E+00	1.82E-01	2.57E-01	
**GSE18229**	**95% HR CI**	1.47 - 10	0.04 - 0.83	0.05 - 1.31	R^2: 0.06
	**p-value**	5.95E-03	2.77E-02	1.02E-01	2.02E-03
	**HR**	4.97E+01	8.58E-01	4.47E+02	
**GSE1456**	**95% HR CI**	6.29 - 393	0.0068 - 108	0.64 - 3.1e+5	R^2: 0.104
	**p-value**	2.12E-04	9.51E-01	6.75E-02	4.01E-04

Table S2. *Superpc continuous prediction results from breast cancer data analysis. The results were generated by using the GSE3143 data set as the training data set and four independent data sets as validation data with a threshold value of 1.20 and 4 selected MCL modules. Since only two modules were selected based on 10-fold cross validation, only two principal components can be used for the superpc analysis. The training data set is highlighted in red. P-values less than 0.05 are highlighted in yellow.*

**Principal Component (PC)**		**1st**	**2nd**	**3rd**	**Overall**
	**HR**	6.45E-01	3.43E+00	6.09E+00	
**NEJM**	**95% HR CI**	0.35 - 1.18	1.24 - 9.48	1.80 - 20.6	R^2: 0.116
	**p-value**	1.54E-01	1.74E-02	3.69E-03	5.40E-08

**GSE4922**	**HR**	3.68E-01	9.04E+00	1.08E+01	
	**95% HR CI**	0.078 - 1.73	1.10 - 74.1	1.31 - 88.9	R^2: 0.056
	**p-value**	2.05E-01	4.03E-02	2.72E-02	1.89E-03
**GSE3143**	**HR**	8.54E+00	1.48E+05	1.54E+08	
	**95% HR CI**	1.42 - 51.3	13 - 1.7e+9	1.1e+4 - 2.2e+12	R^2: 0.142
	**p-value**	5.97E-03	4.22E-04	1.13E-04	8.65E-06
**GSE18229**	**HR**	9.30E-01	7.20E+00	2.48E+00	
	**95% HR CI**	0.49 - 1.78	1.76 - 29.5	0.71 - 8.58	R^2: 0.069
	**p-value**	8.26E-01	6.08E-03	1.53E-01	5.22E-04
**GSE1456**	**HR**	1.81E+00	6.31E+00	1.20E+00	
	**95% HR CI**	0.12 - 26.7	16 - 18449	0.14 - 80	R^2: 0.099
	**p-value**	6.65E-01	4.24E-04	4.60E-01	9.65E-04

Table S3. *Superpc continuous prediction results from breast cancer data analysis. The results were generated by using the GSE18229 data set as the training data set and four independent data sets as validation data with a threshold value of 1.65 and 8 selected MCL modules. The training data set is highlighted in red. P-values less than 0.05 are highlighted in yellow.*

**Principal Component (PC)**		**1st**	**2nd**	**3rd**	**Overall**
**NEJM**	**HR**	2.32E+00	4.00E+00	1.06E+00	
	**95% HR CI**	1.51 - 3.57	2.33 - 6.85	0.49 - 2.28	R^2: 0.119
	**p-value**	1.35E-04	4.70E-07	8.89E-01	2.74E-08
**GSE4922**	**HR**	1.07E+01	6.22E+00	2.86E+00	
	**95% HR CI**	2.50 - 46.3	1.80 - 21.6	0.47 - 17.5	R^2: 0.068
	**p-value**	1.43E-03	3.95E-03	2.56E-01	3.31E-04
**GSE3143**	**HR**	1.20E+02	9.08E+02	1.39E+00	
	**95% HR CI**	9.59 - 1511	22 - 37451	0.45 - 4.24	R^2: 0.081
	**p-value**	2.06E-04	3.32E-04	5.68E-01	2.85E-03
**GSE18229**	**HR**	5.97E+00	1.11E+00	1.64E+00	
	**95% HR CI**	3.13 - 11.4	0.45 - 2.74	0.86 - 3.09	R^2: 0.125
	**p-value**	6.14E-08	8.25E-01	1.30E-01	8.44E-07
**GSE1456**	**HR**	1.00E+01	7.65E+00	2.15E-01	
	**95% HR CI**	1.69 - 59.6	1.22 - 48	0.024 - 1.96	R^2: 0.117
	**p-value**	1.11E-02	2.99E-02	1.73E-01	2.26E-04

Table S4. *Superpc continuous prediction results from breast cancer data analysis. The results were generated by using the GSE1456 data set as the training data set and four independent data sets as validation data with a threshold value of 1.50 and 5 selected MCL modules. The training data set is highlighted in red. P-values less than 0.05 are highlighted in yellow.*

Principal Component (PC)		1st	2nd	3rd	Overall
NEJM	HR	4.39E+00	6.54E-01	4.68E-01	
	95% HR CI	2.49 - 7.73	0.42 - 1.02	0.12 - 1.87	R^2: 0.086
	p-value	3.02E-07	5.84E-02	2.82E-01	3.08E-06
GSE4922	HR	2.77E+01	1.08E+00	1.95E+00	
	95% HR CI	6.01 - 128	0.28 - 4.11	0.035 - 110	R^2: 0.069
	p-value	2.04E-05	9.14E-01	7.46E-01	4.08E-04
GSE3143	HR	2.14E+02	5.23E-01	2.25E-01	
	95% HR CI	0.23 - 2.0e+5	0.06 - 4.76	3.9e-4 - 128	R^2: 0.02
	p-value	1.25E-01	5.65E-01	6.45E-01	3.18E-01
GSE18229	HR	4.39E+00	4.27E-01	1.10E+00	
	95% HR CI	1.75 - 11	0.22 - 0.83	0.24 - 5.09	R^2: 0.055
	p-value	1.57E-03	1.16E-02	9.06E-01	2.23E-03
GSE1456	HR	1.86E+02	3.10E+00	6.58E+00	
	95% HR CI	20 - 1782	0.48 - 20	0.04 - 1083	R^2: 0.129
	p-value	5.65E-06	2.37E-01	4.69E-01	7.52E-05

In order to check the consistence of results using different data set as the training data set, we did an overlapping analysis of gene sets collected from selected MCL modules from the trained super models. Table S5 shows results from this analysis, and p-values were calculated based on hyper-geometric test.

Table S5. *Gene set overlapping analysis for genes collected from selected MCL modules from trained superpc models. Names in the first column and the first row are data sets used as the training data sets.*

	NEJM (165)	GSE4922 (229)	GSE3143 (47)	GSE18229 (136)	GSE1456 (117)
NEJM (165 genes)		47 (3.74E-38)	3 (0.048)	20 (1.69E-13)	28 (1.22E-24)
GSE4922(229 genes)			1 (0.69)	38 (1.74E-30)	66 (1.70E-78)
GSE3143(47 genes)				11 (4.08E-11)	0 (1.0)
GSE18229(136 genes)					19 (2.96E-15)
GSE1456(117 genes)					

Comparison results from the superpc analysis using network modules generated from weighted FI network or un-weighted FI network

Table s6. *Superpc analysis results from network modules from the weighted FI network or un-weighted FI network. R^2 and p-values were results from the trained superpc model using NEJM (highlighted in red) as the training data set.*

		Weighted FI	Un-weighted FI
NEJM	R^2	0.16	0.129
	p-value	6.82E-11	2.30E-09
GSE4922	R^2	0.072	0.066

	**p-vale**	1.86E-04	4.67E-04
**GSE3143**	**R^2**	0.059	0.027
	**p-value**	1.56E-02	2.16E-01
**GSE18229**	**R^2**	0.082	0.072
	**p-value**	4.71E-05	5.06E-04
**GSE1456**	**R^2**	0.131	0.099
	**p-value**	7.46E-05	9.98E-04

Comparison of the MCL+superpc algorithm to gene based superpc and greedy network component signature discovery algorithm

Our signature identification method uses MCL to identify candidate disease-related network modules, and superpc to build prognostic signatures from those modules. We asked what contribution the MCL clustering step makes to the overall strength and robustness of the resulting signatures. To do this, we compared our method to superpc using individual genes, as well as to superpc applied to modules discovered by a popular greedy algorithm. The former comparison eliminates network topology information entirely from the discovery phase; the latter uses network information, but not MCL clustering. In both comparisons, we used each of 5 breast cancer data sets for training data, and the remaining four data sets for validation

Gene-based superpc yielded significant trained superpc models regardless which data set was used as the training data set (data not shown). However, if we focus our attention to the most significant principal component in the training data set, the results from the network-clustering approach are more consistent across the validation sets than the gene-based approach (Figure S1). For example, the first PC from the model using GSE18229 as the training data set is not significant for the GSE4922 and GSE1456 data sets (Figure S1B), but remains significant in the MCL+superpc approach (Figure S1A).

The most widely cited network-based prognostic signature search algorithm was developed by Ideker et al (Ideker et al 2002), which is a greedy search algorithm. We compared our approach to the greedy algorithm, and found that our method runs much faster: about 20 seconds versus 6 hours for an implementation of the greedy search algorithm. Since the greedy search method uses clinical information directly during network module finding, we might expect overtraining issues, and indeed we observed evidence of this. Table S7 shows the results of using network modules discovered by greedy search for superpc analysis using the GSE4922 as the training data set. There is far more variability in the p-values resulting from the greedy approach among the training and testing data sets, than in the p-values obtained from modules discovered by MCL. For example, the difference of p-values between GSE4922, the training data set, and Nejm is greater than 10 orders of magnitude, while the difference in the MCL based approach is only 4 orders of magnitude. It is interesting to notice that the Nejm data set yielded lowest p-value when GSE4922 was used as the training

data set in the MCL approach (Table S1), but not in the greedy search approach. Similarly, network modules identified by greedy search exhibited much wider 95% confidence intervals of hazard ratios across all five breast cancer data sets than MCL modules (Tables S1, S7).

Table S7. *Superpc analysis results using network modules from the greedy search algorithm. The result were generated from using the GSE4922 data set as the training data set, and other four data sets as the testing data sets, with a cutoff value at 2.25 for superpc.*

**Principal Component (PC)**		**1st**	**2nd**	**3rd**	**Overall**
**NEJM**	**HR**	7.06E+01	8.06E-01	1.97E+00	
	**95% HR CI**	12.5 - 398	0.06 - 11.4	0.096 - 40.5	R^2: 0.089
	**p-value**	1.40E-06	8.73E-01	6.61E-01	5.86E-06
**GSE4922**	**HR**	6.52E+07	1.61E+13	1.89E+19	
	**95% HR CI**	9.4e+5 - 4.5e+9	9.1e+7 - 2.9e+18	3.7e+12 - 9.7e+25	R^2: 0.398
	**p-value**	<2E-16	8.12E-07	1.82E-08	0.00E+00
**GSE3143**	**HR**	7.54E+04	5.03E+10	1.33E-02	
	**95% HR CI**	0.9 - 6.4e+9	2.8 - 9.0e+20	5.3e-8 - 3.3e+3	R^2: 0.044
	**p-value**	5.25E-02	4.07E-02	4.96E-01	7.74E-02
**GSE18229**	**HR**	1.56E+01	5.60E-03	1.70E-01	
	**95% HR CI**	0.92 - 263	7.8e-5 - 0.40	1.7e-3 - 17	R^2: 0.05
	**p-value**	5.70E-02	1.76E-02	4.52E-01	6.80E-03
**GSE1456**	**HR**	3.55E+07	1.18E+01	3.00E+07	
	**95% HR CI**	2.1e+4 - 6.0e+10	1.0e-7 - 1.4e+9	8.7e-4 - 1.0e+18	R^2: 0.13
	**p-value**	4.62E-06	7.95E-01	1.64E-01	9.68E-05

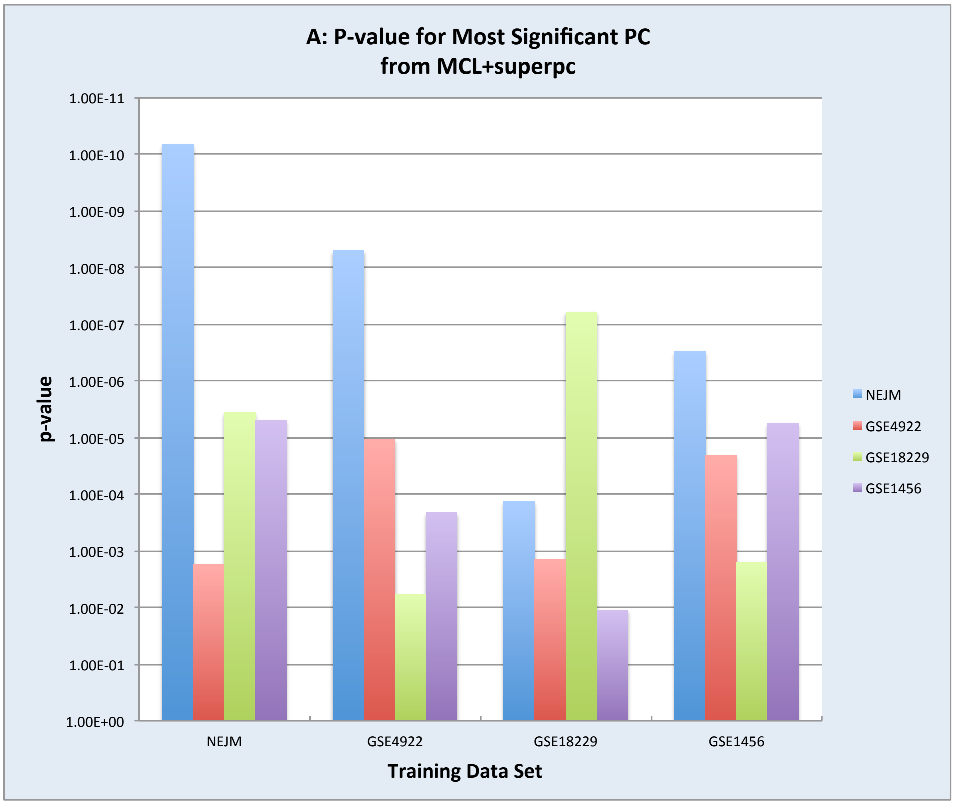

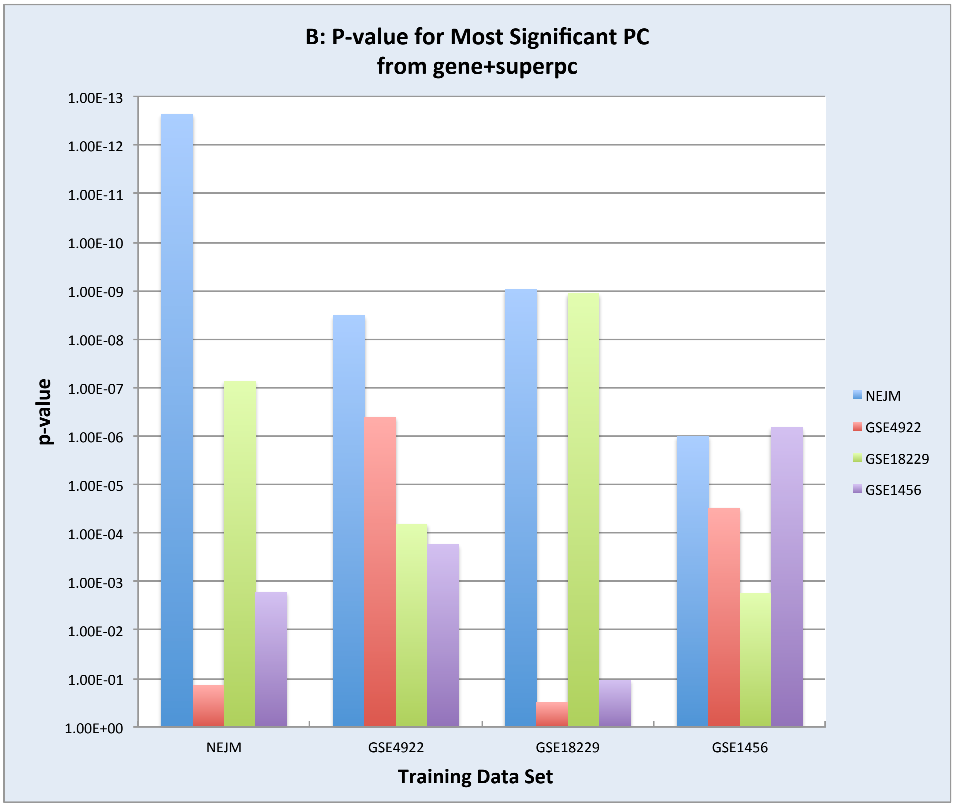

Figure S1. *P-values for the most significant principal component from the superpc analysis. A: results from the MCL module based superpc approach. B: results from the gene based superpc analysis.*

Box plot of module 2 gene expression scores

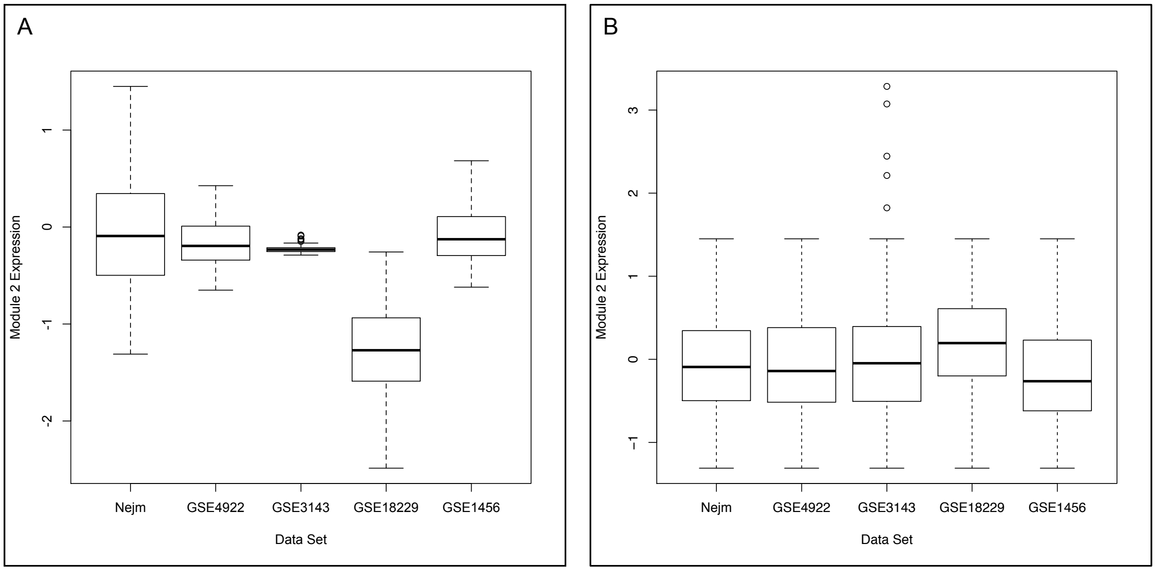

Figure S2. *Box plot of module 2 gene expression scores. In order to compare and merge all breast cancer data set together, we rescaled module 2 expression scores across the four validation data sets so that they share the same mean and 95th percentile ranges (top and bottom bars in the box plots below). A: Box plot of module 2 gene expression for five breast cancer data sets. B: Box plot of module 2 gene expression after values in four validation data sets are re-scaled based on the Nejm module 2 expression distribution.*

Kaplan-Meier survival plot for 4 groups of breast cancer patients based on the GSE18229 data set.

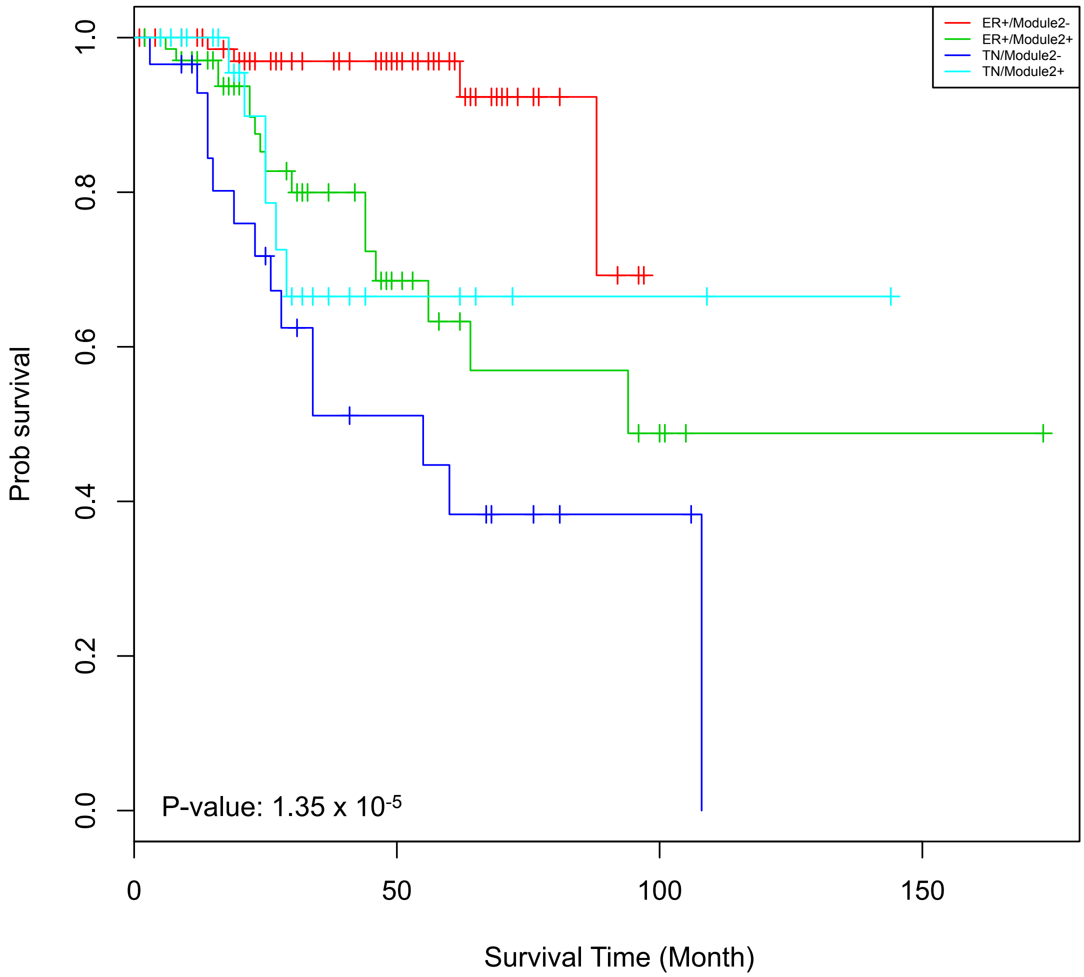

Figure S3. *Kaplan-Meier survival plot for 4 groups of breast cancer patients based on the GSE18229 data set. ER+ samples, and triple negative (TN) samples are divided into two groups separately based on module 2 expression median values.*

Functional annotations of breast cancer module 2 based on pathways and GO annotations

Table S8. *Functional annotations of module 2 based on pathways (rows 1 – 3), GO biological process terms (rows 4 – 9), and GO cellular component terms (rows 9 – 14).*

Gene Set Name	Gene Set Size	Module 2 Genes in Gene Set	P-value	FDR	Module 2 Gene Names in Gene Set
M Phase	96	26	0	<5.00e-04	ITGB3BP,MAD1L1,CCDC99,KNTC1,AURKB,SPC25,CDCA8,INCENP,BUB1,ZWILCH,NUDC,ZW10,ERCC6L,CENPN,TAOK1,CENPQ,BIRC5,CENPE,NDC80,CENPI,CENPH,MAD2L1,RCC2,SEC13,BUB1B,KIF20A
Aurora B signaling	39	11	0	<5.00e-04	AURKC,AURKB,CDCA8,NCAPG,INCENP,BUB1,NSUN2,BIRC5,NDC80,NCAPD2,KIF20A

Signaling by Aurora kinases	94	11	0	<3.33e-04	AURKC,AURKB,CDCA8,NCAPG,INCENP,BUB1,NSUN2,BIRC5,NDC80,NCAPD2,KIF20A
mitosis	138	15	0	<3.333e-04	KNTC1,AURKB,SPC25,CDCA8,NCAPG,INCENP,BUB1,ZWILCH,NUDC,ERCC6L,BIRC5,NCAPD2,MAD2L1,RCC2,BUB1B
cell division	169	17	0	<3.333e-04	MAD1L1,KNTC1,SPC25,CDCA8,NCAPG,BUB1,ZWILCH,NUDC,ZW10,ERCC6L,CENPE,NDC80,CENPH,NCAPD2,MAD2L1,RCC2,BUB1B
cell cycle	297	19	0	<3.333e-04	MAD1L1,AURKC,KNTC1,AURKB,SPC25,CDCA8,NCAPG,INCENP,BUB1,ZWILCH,NUDC,ZW10,ERCC6L,BIRC5,CENPE,NDC80,NCAPD2,MAD2L1,RCC2
mitotic cell cycle checkpoint	10	6	0	<2.500e-04	MAD1L1,KNTC1,ZWILCH,ZW10,MAD2L1,BUB1B
cytokinesis	26	4	0	<2.000e-04	AURKC,AURKB,INCENP,BIRC5
mitotic metaphase	2	2	0	6.67E-04	MAD1L1,CENPE
chromosome, centromeric region	27	10	0	<1.00e-03	ITGB3BP,AURKB,CDCA8,ERCC6L,CENPN,CENPQ,BIRC5,NDC80,CENPI,RCC2
condensed chromosome kinetochore	32	9	0	<5.00e-04	KNTC1,SPC25,BUB1,ZWILCH,ZW10,ERCC6L,CENPN,NDC80,CENPH
chromosome	114	8	0	<3.33e-04	ITGB3BP,AURKB,CDCA8,CENPN,CENPQ,BIRC5,CENPI,CENPH
spindle	46	5	0	<2.50e-04	MAD1L1,AURKB,INCENP,CENPE,RCC2
nucleus	3401	24	0	<2.00e-04	ITGB3BP,MAD1L1,AURKC,KNTC1,AURKB,SPC25,CDCA8,NCAPG,INCENP,BUB1,NSUN2,NUDC,ZW10,CENPN,CENPQ,BIRC5,CENPE,NDC80,CENPI,CENPH,NCAPD2,MAD2L1,RCC2,BUB1B

Survival analysis results for 31 genes contained by module 2

Table S9. *Survival analysis results for 31 genes contained by module 2 using univariate Cox proportional hazards model. Genes that are significant across all five breast cancer data sets have been highlighted in yellow using p-value cutoff = 0.05. The genes are sorted by p-values based on the Nejm data set. Empty cells are for genes that couldn't be mapped in the listed data set.*

Gene	Nejm		GSE4922		GSE3143		GSE18229		GSE1456	
	Coefficient	p-value	Coefficient	p-value	Coefficient	p-value	Coefficient	p-value	Coefficient	p-value
**BIRC5**	3.37E-01	2.15E-10	9.78E-01	2.23E-04	4.86E+00	5.20E-03	3.26E-01	1.92E-03	1.26E+00	4.83E-04
**AURKB**	5.23E-01	7.99E-10	8.35E-01	5.79E-04	2.36E+00	1.25E-07	8.15E-01	6.46E-05	1.25E+00	1.77E-03
**KIF20A**	4.32E-01	8.77E-10	1.21E+00	7.04E-05			4.97E-01	9.38E-04	1.66E+00	8.72E-04
**NCAPD2**	4.71E-01	7.01E-09	2.72E-01	5.27E-01	3.55E+00	1.07E-01	8.70E-01	2.52E-03	6.68E-01	3.27E-01
**CENPN**	4.22E-01	1.54E-08	7.46E-01	1.35E-04			4.04E-01	8.06E-03	1.70E+00	5.99E-05
**BUB1**	4.27E-01	1.77E-08	5.84E-01	1.99E-01	1.04E+00	7.55E-01	4.89E-01	2.45E-04	1.40E+00	8.30E-03
**SPC25**	4.20E-01	4.50E-08	9.02E-01	1.52E-02	-2.12E+00	5.02E-01	5.11E-01	1.14E-02	8.71E-01	4.53E-02
**RCC2**	8.25E-01	7.92E-07					3.73E-01	1.83E-01	1.89E+00	4.71E-03
**CDCA8**	4.72E-01	9.05E-07	5.24E-01	3.69E-05			2.78E-01	9.48E-02	6.08E-01	1.89E-03
**MAD2L1**	3.71E-01	2.37E-06	7.91E-01	2.18E-04	-1.27E+00	7.14E-01	2.52E-01	4.10E-02	1.15E+00	8.10E-04
**NSUN2**	6.49E-01	4.72E-06					3.05E-01	1.98E-01	1.55E+00	1.83E-02
**SEC13**	6.20E-01	1.98E-05	8.07E-01	1.51E-01	7.31E-01	3.30E-01	8.04E-01	7.07E-02	1.72E+00	1.38E-02
**CCDC99**	6.69E-01	2.28E-05	9.48E-01	1.02E-02			9.52E-01	2.29E-05	6.21E-01	1.21E-01
**NDC80**	3.83E-01	4.54E-05	5.43E-01	3.10E-03	3.04E+00	3.69E-02	2.52E-01	1.72E-01	7.21E-01	1.08E-02

**BUB1B**	5.33E-01	6.51E-05	1.36E+00	9.22E-05	3.74E+00	3.35E-02	2.50E-01	1.70E-01	1.93E+00	9.14E-05
**ANKZF1**	4.12E-01	1.50E-03	-2.55E-01	2.67E-01			6.41E-01	5.39E-03	5.25E-02	8.41E-01
**KNTC1**	4.07E-01	2.13E-03	1.30E-01	7.55E-01	1.65E+00	5.95E-01	2.58E-01	1.97E-01	1.30E+00	1.77E-02
**ZWILCH**	4.66E-01	1.84E-02	1.25E+00	4.53E-03			6.88E-01	1.21E-02	1.42E+00	1.57E-02
**CENPE**	1.95E-01	3.71E-02	5.90E-01	1.17E-04	2.93E+00	4.08E-01	6.33E-01	2.95E-04	9.12E-01	1.74E-04
**ZW10**	3.52E-01	4.90E-02	-8.63E-02	9.04E-01	-4.41E+00	2.15E-01	7.81E-01	6.27E-03	3.62E-01	7.53E-01
**CENPQ**	2.77E-01	5.84E-02	-4.24E-02	9.30E-01			1.56E-01	4.83E-01	-1.19E-01	8.54E-01
**MAD1L1**	3.92E-01	6.70E-02	1.01E+00	4.65E-03	5.22E+00	8.49E-03	7.91E-02	7.63E-01	6.13E-01	1.49E-01
**ERCC6L**	1.21E-01	7.38E-02							9.88E-01	1.60E-03
**AURKC**	2.77E-01	1.45E-01	1.48E-01	7.84E-01	4.48E+00	1.71E-02	-4.11E-02	8.95E-01	1.24E+00	1.10E-01
**CENPH**	2.52E-01	1.62E-01					4.04E-01	5.59E-02	9.21E-01	3.14E-02
**NUDC**	2.10E-01	2.51E-01	7.79E-01	2.34E-01	1.08E+00	2.58E-01	-2.21E-01	4.95E-01	2.02E+00	9.80E-03
**INCENP**	-2.38E-01	3.04E-01	5.01E-02	7.97E-01			3.06E-01	2.27E-02	1.01E+00	1.66E-02
**NCAPG**	1.18E-01	3.14E-01	1.04E+00	1.07E-04			3.31E-01	7.12E-02	1.71E+00	2.28E-04
**CENPI**	1.47E-01	3.39E-01	6.05E-01	6.02E-02	1.33E+00	6.82E-01	7.83E-01	1.07E-04	1.45E+00	1.08E-03
**TAOK1**	1.61E-01	4.53E-01	-2.96E-01	4.66E-01			-4.07E-01	1.09E-01	-9.26E-01	8.16E-02
**ITGB3BP**	-2.41E-02	8.51E-01	1.14E+00	5.75E-02	8.46E-01	7.42E-01	-3.24E-02	8.93E-01	4.46E-01	5.81E-01

Relationship between module 2 and conventional breast cancer prognostic factors

A number of variables are conventionally used to predict patient prognosis in breast cancer, including anatomic factors such as lymph node status and molecular biomarkers such as tumor estrogen receptor status. To determine the relationship between module 2 gene expression level and these conventional prognostic factors, we downloaded lymph node status (Posnodes), estrogen receptor alpha expression (ESR1), patient clinical characteristics according to the NIH scale (NIH), and patient clinical characteristics according to St. Gallen criteria (StGallen). These factors, along with module 2 expression levels, were entered into a regression model using a multi-covariate Cox hazards model. As shown in Table S10, module 2 expression still contributes significantly to patient survival after adjusting for these four variables (HR 2.74, 95% CI 1.70-4.39), although the p-value has increased to 3.2×10^{-5}, indicating that module 2 expression is correlated with at least one of these four clinical factors.

To understand the relationship among module 2 expression and the four clinical variables in more detail, we performed a two-variable Cox hazards analysis followed by a stratified Cox analysis on each clinical variable (Table S11), as well as a direct correlation analysis between module 2 and the variables (Table S12). These analyses identified a strong anti-correlation between module 2 expression and ESR1 positivity. After stratification for ESR1, we found that module 2 expression was strongly predictive of poorer prognosis in ESR1 positive patients (hazard ratio 4.70; 95% CI 2.64-8.38; p-value = 1.51×10^{-7}; N=226), but was not statistically significant at predicting survival in ESR1 negative patients (hazard ratio 1.47; 95% CI 0.75 – 2.88; p-value = 0.26; N=69). As might be expected the NIH and StGallen criteria are anti-correlated with increasing module 2

expression. Similar results were found in each of the four validation series (data not shown).

Table S10. *Survival analysis results based on multi-covariate Cox proportional hazards model using module 2 expression and four clinical variables from the Nejm data set.*

Variable	Coefficient	P-value	Hazard Ratio (HR)	95% CI of HR
Module 2 Expression	1.01	3.16×10^{-5}	2.74	1.70 - 4.39
ESR1	-0.5	0.066	0.6	0.35 - 1.03
NIH	-0.62	0.54	0.54	0.072 - 4.01
StGallen	-1.08	0.3	0.34	0.044 - 2.60
Posnodes	-0.18	0.44	0.83	0.53 - 1.32

Table S11. *Survival analysis results based on stratified Cox proportional hazards models. The models were constructed by stratifying on the variables listed in the first column. The last row shows results from uni-variate Cox model for reference (see Table 3 in the main text).*

Stratified Covariate	Coefficient	P-value	Hazard Ratio (HR)	95% CI of HR
ESR1	1.08	5.76×10^{-6}	2.96	1.85 – 4.73
NIH	1.3	2.99×10^{-10}	3.65	2.44 – 5.47
StGallen	1.23	4.01×10^{-9}	3.43	2.27 – 5.16
Posnodes	1.29	2.23×10^{-10}	3.65	2.45 – 5.44
(Module 2 Expression)	1.3	1.75×10^{-10}	3.68	2.47 - 5.49

Table S12. *Correlation analysis between module 2 expression and three other variables: ESR1, NIH and StGallen. In the T-test, samples were divided into two groups based on co-variate values.*

Covariate	Pearson Correlation			T-test		
	Correlation	95% CI	P-value	Mean in Group 1	Mean in Group 2	P-value
ESR1	-0.47	-0.55 -0.37	$< 2.2 \times 10^{-16}$	0.42	-0.19	9.34×10^{-14}
NIH	-0.13	-0.24 -0.019	0.022	-0.031	-0.41	0.052
StGallen	-0.26	-0.36 -0.15	8.07×10^{-6}	-0.0054	-0.54	6.86×10^{-7}

Relationship between module 2 and breast cancer sub-types from microarray gene expression classifier

Using the PAM50 classifier (Parker et al 2009) (R code downloaded from https://genome.unc.edu/pubsup/breastGEO/), we classified samples in the Nejm data set into five subtypes Luminal A, Luminal B, Basal, Her2, and Normal-like. As reported previously (Sorlie et al 2003; Hu et al 2006; Ivshina et al 2006), we saw that Luminal A samples had better overall survival times, and the Basal subtype had the worst (Figure S4A).

We examined the relationship between breast cancer subtypes and module 2 expression (Figure S4B). High module 2 expression is positively correlated with the Basal and Her2 subtypes both associated with more aggressive disease, and negatively correlated with the Luminal A subtype. Multi-covariate Cox-PH analysis indicates that Module 2 independently contributes to patient survival in the NEJM (HR 1.79, 95% CI of HR 1.00-3.19, p-value 0.049), GSE3143 (p-value 1.02×10^{-3}) and GSE4922 (p-value 3.71×10^{-3}). Module 2 failed to achieve statistical significance in the multi-covariate Cox-PH analysis for the GSE1456 and GSE18229 data sets.

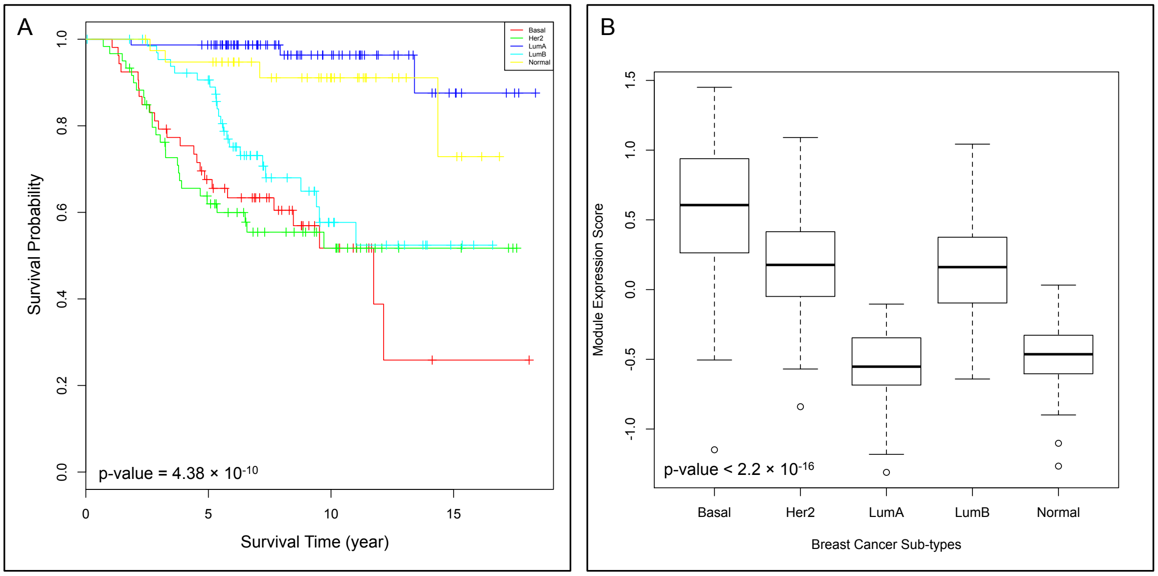

Figure S4. *A: Kaplan-Meier survival plot for the Nejm data set after classifying samples into subtypes based on the PAM50 classifier (Parker et al 2009). Curves for samples in different subtypes are drawn in different colors. B: Box plot of module 2 expression in five breast cancer sub-types for the Nejm data set. P-value was calculated based on ANOVA.*

Comparison between Module 2 and other breast cancer prognostic gene signatures

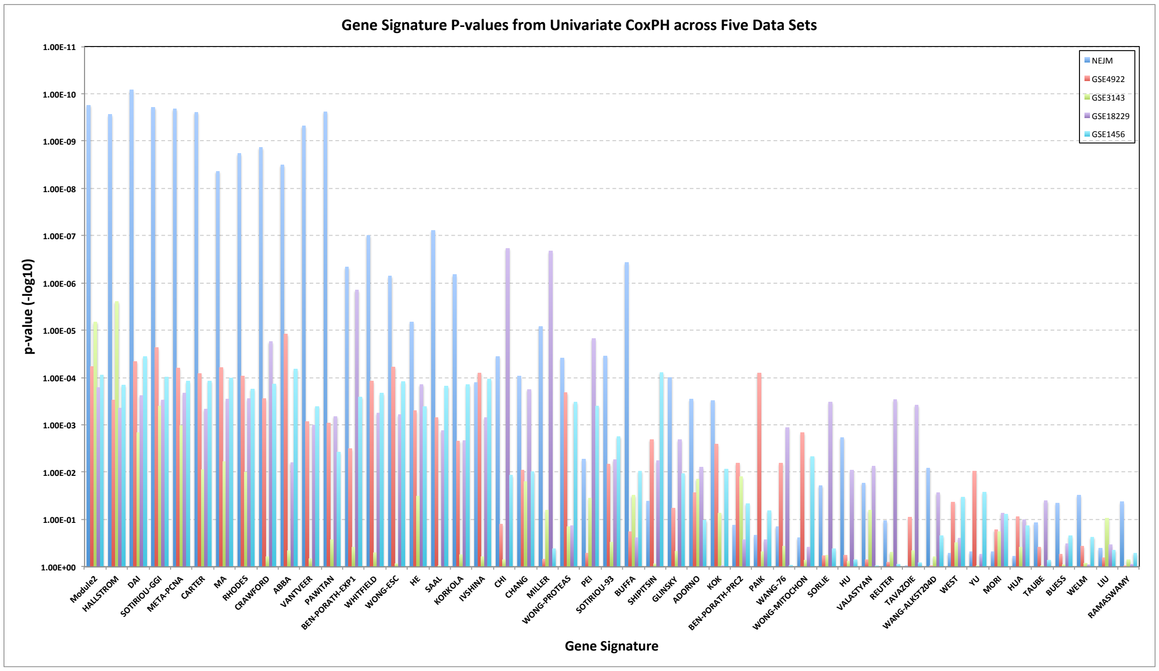

Figure S5. *Plot of p-values from univariate CoxPH survival analyses using five breast cancer data sets for module 2 and 48 published breast cancer gene signatures.*

Table S13. *Pearson correlation coefficients between module 2 gene expression scores and meta-PCNA gene expression scores in five breast cancer data sets.*

Data Set	Correlation	p_value
**NEJM**	0.97	6.8E-175
**GSE4922**	0.97	3.2E-155
**GSE3143**	0.8	8.5E-37
**GSE18229**	0.98	2.3E-253
**GSE1456**	0.96	9.6E-89

Table S14. *Multi-covariate CoxPH survival analysis results for module 2 and meta-PCNA gene set. Cells for p-values less than 0.05 are highlighted in yellow.*

Data Set	Gene Set	HR	95% CI	P-value	Overall P-value
	**Meta-PCNA**	2.02	0.48 - 8.46	0.34	
**NEJM**	**Module 2**	1.85	0.43 - 7.98	0.41	1.20E-09
	**Meta-PCNA**	2.75	0.03 - 300	0.67	
**GSE4922**	**Module 2**	2.73	0.05 - 149	0.62	2.90E-04
	**Meta-PCNA**	0.87	7.8E-4 - 9.8E+2	0.97	
**GSE3143**	**Module 2**	6.50E+06	8.5E+1 - 5.0E+11	6.20E-03	3.90E-05
	**Meta-PCNA**	0.86	0.06 - 13.3	0.91	
**GSE18229**	**Module 2**	3.48	0.30 - 39.8	0.32	8.30E-04
	**Meta-PCNA**	11.2	0.05 - 2738	0.39	
**GSE1456**	**Module 2**	2.03	0.03 - 136	0.74	5.10E-04

Application of the MCL+superpc approach to high-grade serous adenocarcinoma of the ovary

Table S15. Superpc continuous prediction results by choosing the TCGA ovarian cancer data set as the training data set, and the threshold value of 0.8. P-values less than 0.05 are highlighted in yellow. The first column shows the data sets, TCGA, the training data set, is highlighted in red.

**Principal Component (PC)**		**1st**	**2nd**	**3rd**	**Overall**
**TCGA**	**HR**	1.04E+00	1.32E+00	1.16E+00	
	**95% HR CI**	0.93 - 1.16	1.14 - 1.53	1.77 - 0.077	R^2: 0.035
	**p-value**	5.37E-01	2.81E-04	7.66E-02	9.19E-04
**GSE9891**	**HR**	2.44E+00	3.96E+00	6.85E-01	
	**95% HR CI**	1.08 - 5.54	1.09 - 14.4	0.25 - 1.88	R^2: 0.048
	**p-value**	3.28E-02	3.64E-02	4.64E-01	8.26E-03
**GSE13876**	**HR**	1.46E+00	1.53E+01	7.16E-01	
	**95% HR CI**	0.56 - 3.79	3.70 - 63	0.26 - 1.99	R^2: 0.105
	**p-value**	4.39E-01	1.65E-04	5.23E-01	8.10E-04
**GSE26712**	**HR**	8.35E-01	5.87E+00	2.08E+00	
	**95% HR CI**	0.29 - 2.42	1.35 - 25	0.64 - 6.78	R^2: 0.045
	**p-value**	7.40E-01	1.81E-02	2.23E-01	3.48E-02

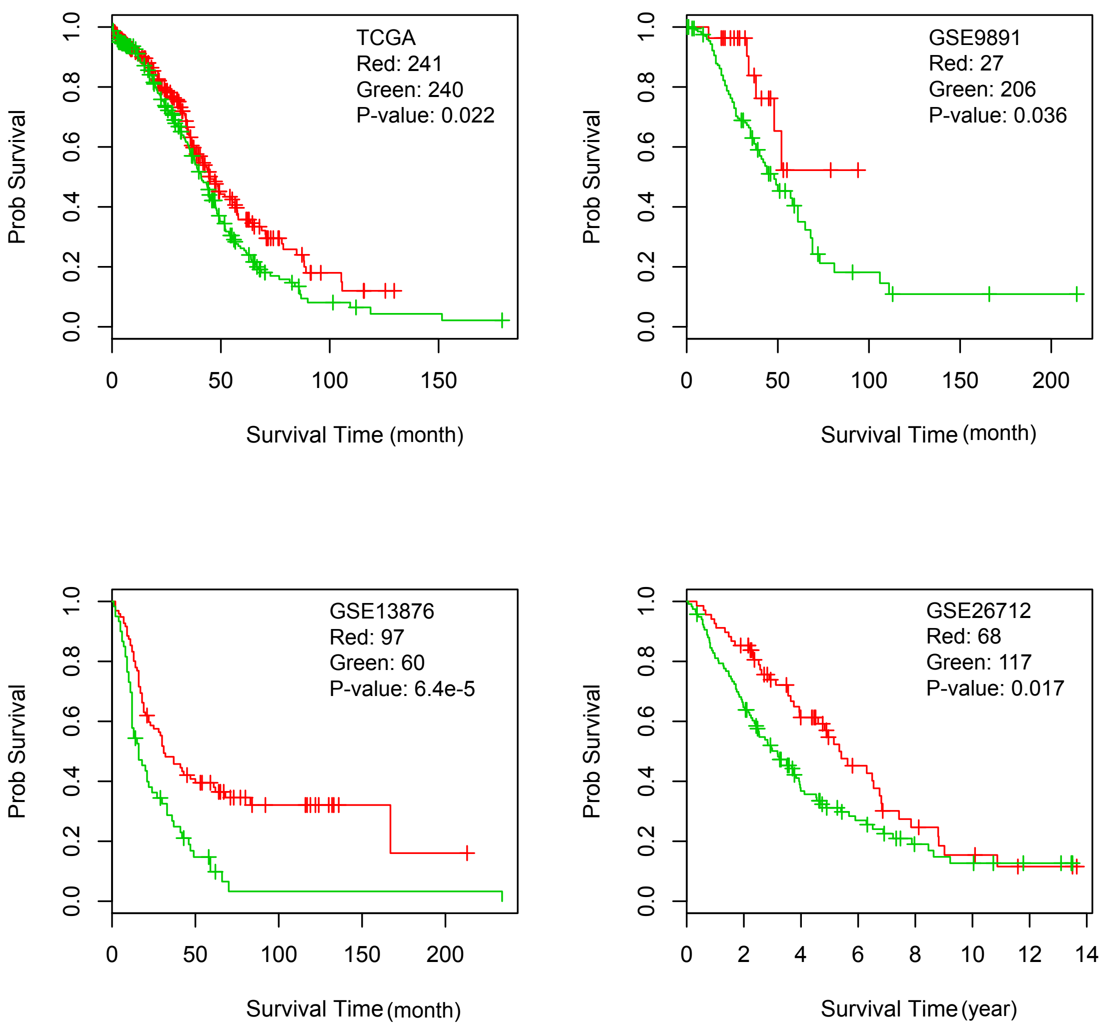

Figure S6. *Kaplan-Meier survival analysis of the ovarian cancer data sets based on the superpc discrete prediction. The discrete prediction used the TCGA data set as the training data set, and the second principal component as feature.*

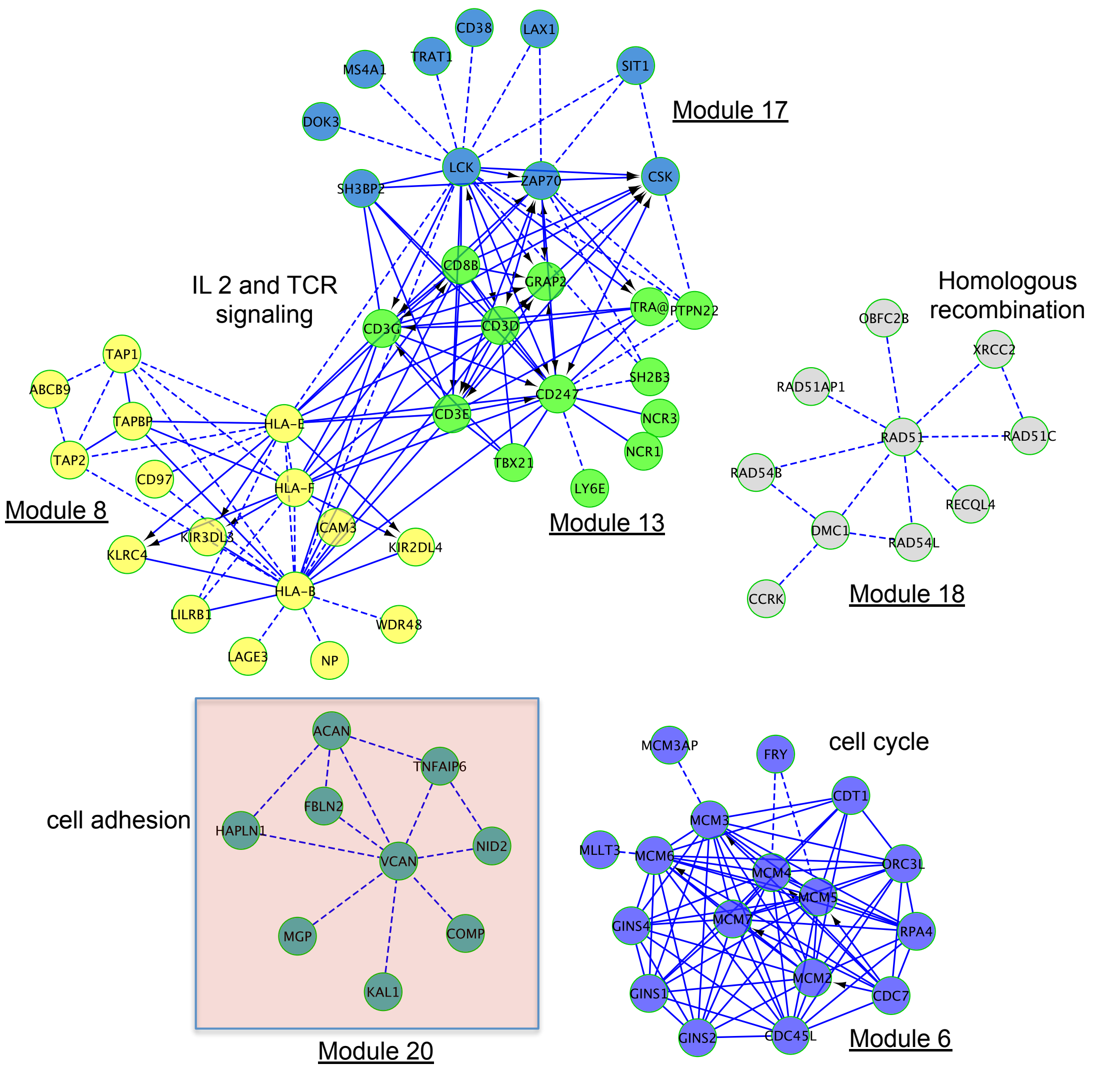

Figure S7. *FI sub-network constructed by genes contained by modules from the TCGA ovarian cancer data set. The top 6 MCL modules from the TCGA ovarian cancer data set were selected by the superpc analysis. Modules are labeled based on the order generated by module sizes. Modules 8, 13 and 17 enrich genes related to IL2 and TCR signaling pathways. Modules 6, 18, and 20 enrich genes related to cell cycle, homologous recombination, and cell adhesion, respectively. CoxPH analysis results show that except module 20, all other modules in the diagram have negative hazard ratios.*

References

Hu, Z., Fan, C., Oh, D.S., Marron, J.S., He, X., Qaqish, B.F., Livasy, C., Carey, L.A., Reynolds, E., Dressler, L., Nobel, A., Parker, J., Ewend, M.G., Sawyer, L.R., Wu, J., Liu, Y., Nanda, R., Tretiakova, M., Orrico, A.R., Dreher, D., Palazzo, J.P., Ivshina, A.V., George, J., Senko, O., Mow, B., Putti, T.C., Smeds, J., Lindahl, T., Pawitan, Y., Hall, P., Nordgren, H., Wong, J.E.L., Liu, E.T., Bergh, J., Kuznetsov,

V.A. & Miller, L.D. *Genetic reclassification of histologic grade delineates new clinical subtypes of breast cancer*. Cancer Res, **2006**, Vol. 66(21), pp. 10292-10301

Ideker, T., Ozier, O., Schwikowski, B. & Siegel, A.F. (2002) Discovering regulatory and signalling circuits in molecular interaction networks. Bioinformatics 18 Suppl 1: S233–S240.

Parker, J.S., Mullins, M., Cheang, M.C.U., Leung, S., Voduc, D., Vickery, T., Davies, S., Fauron, C., He, X., Hu, Z., Quackenbush, J.F., Stijleman, I.J., Palazzo, J., Marron, J.S., Nobel, A.B., Mardis, E., Nielsen, T.O., Ellis, M.J., Perou, C.M. & Bernard, P.S. *Supervised risk predictor of breast cancer based on intrinsic subtypes*. J Clin Oncol, **2009**, Vol. 27(8), pp. 1160-1167

Sørlie, T. *Molecular portraits of breast cancer: tumour subtypes as distinct disease entities*. Eur J Cancer, **2004**, Vol. 40(18), pp. 2667-2675
